# Can the training regimen influence night time physical activity in racehorses?

**DOI:** 10.1016/j.vas.2021.100208

**Published:** 2021-09-25

**Authors:** Aires Santana Rumpel, Marcelo Meller Alievi, José Osvaldo Jardim Filho, Cesar Augusto Camacho Rozo, Lucas Antonio Heinen Schuster, Alessandra Ventura da Silva, Márcio Poletto Ferreira

**Affiliations:** a Graduate Program in Animal Medicine: Equine, Federal University of Rio Grande do Sul, Porto Alegre, RS, Brazil; b Department of Animal Medicine, Federal University of Rio Grande do Sul, Porto Alegre, RS, Brazil; c Department of Large Animal Clinics, Federal University of Santa Maria, Santa Maria, RS, Brazil

**Keywords:** Accelerometer, Equine, Ethology, Locomotor activity, Rest, Training protocol

## Abstract

Physical activity has been widely investigated in horses to elucidate locomotion characteristics and behavior. However, research in real environment of training stables is limited. The objective of this study was to evaluate the effect of training regimen on night time physical activity of racehorses. Physical activities of twenty animals were monitored during the night time using accelerometers. The animals were compared in terms of training regimen: horses subjected to training on continual days, and horses subjected to training on intermittent days; age and sex were also considered. The variables analyzed were: counts per minute, percentage of time in (sedentary, light, moderate and vigorous) physical activity. Statistical analysis was performed by the PROCGLM procedure (ANOVA) and the means were calculated by Tukey's test. The training regimen impacts the physical activity of thoroughbreds. The mean counts per minute showed a higher physical activity in the intermittent trained animals in relation with continuously trained animals. The continuously trained animals presented a greater percentage of time in sedentary physical activity than those intermittent trained. However, intermittently trained horses spent a larger percentage of time in moderate and vigorous physical activity, in comparison with continuously trained animals. In conclusion, racehorses subject to training on continual days have lower physical activity in the night time than those that train on intermittent days.

## Highlights

• The training regimen influenced the night time physical activity of athlete horses.
• Thoroughbred racing horses trained on continual days had lower physical activity in the night time than those trained on intermittent days.
• Accelerometry is a good tool for measuring horse movements in box stalls, and there is an area for further studies with the use of accelerometers in equines.

## Introduction

Locomotor activity in horses is influenced by different factors, such as photoperiod (Bertolucci, Giannetto, Fazio & Piccione, 2008), feeding management (Giannetto, Fazio, Alberghina, Panzera & Piccione, 2015), rearing system (in the field or stable) (Piccione, Costa, Gianneto & Caola, 2008), and paddock size (Maisonpierre et al., 2019). Horses are animals which, in liberty, have day time and night time feeding habits, spend 46% to 66% of their time feeding (Boyd, Carbonaro & Houpt, 1988; Duncan, 1985), with circadian and seasonal variations depending on climatic conditions and food quality (Auer, Kelemen, Engl & Jenner, 2021). When these animals are stabled for training, they spend less time for food intake, and this time is taken up by other normal (standing alert, observing, relaxing, sleeping, urinating, defecating, seeking contact, interacting) or undesirables (restlessness, head tossing/nodding, box walking, licking/food searching, attacking, apathetic/depressed, cribbing, coprophagy) activities (Ribeiro, Matzkeit & Nicolau, 2019), which may compromise physical performance the next day (McGreevy, 2004). Thoroughbred horses, which are more nervous, energetic, poignant, and rebellious, have, on average, higher locomotor activity than other breeds such as Standardbred and the Italian saddle horse (Giannetto et al., 2016). They have a locomotor activity peak in the middle of the day (Piccione et al., 2008), a more frequent locomotor activity in spring and fall (Bertolucci et al., 2008) and their physical activity increases with confinement (Giannetto et al., 2016; Piccione et al., 2008).

Confined mares, which do not exercise every day, show compensatory increase in locomotion when let out of confinement, indicating a response to exercise deprivation (Houpt, Houpt, Johnson, Erb & Yeon, 2001). Levels of activity in horses have been evaluated through the use of accelerometry, identifying cut points in the level of activity, classifying activities into: sedentary, light, moderate, and vigorous (Morrison et al., 2015). In human and animal studies, accelerometry is validated against directly observed movement or against energy expenditure, both methods being regarded widely as “gold standards” for the validation of movement (De Vries et al., 2009; Yam et al., 2011). In humans, has been shown that there is an influence of gender and age on physical activity (Belcher et al., 2010; Troiano et al., 2008), as well as a contribution of training in athletes (Yoshida et al., 2014).

Therefore, it is important to map out the physical activity of horses when they are alone closed in their box stalls, to understand the influence of training regimen on physical activity in the night time and so assist in the development of more appropriate training strategies that might improve the welfare and performance. Thus, the aim of this study was to evaluate the effect of training regimen on night time physical activity of racehorses, considering the influence of age and sex. The hypothesis of this work was that horses subjected to training on continual days would have lower physical activity in the night time, compared to horses subjected to training on intermittent days.

## Materials and methods

### Animals and sites

Ten thoroughbred racehorses that trained on intermittent days and ten that trained on continual days were randomly selected of the same stable. The number of twenty horses was defined according to previous studies (Giannetto et al., 2016; Maisonpierre et al., 2019). The experiment was conducted at the Jockey Club do Rio Grande do Sul, Porto Alegre, Brazil (30° 05′ 20.1″ S, 51° 14′ 50.5″W). All animals were previously subjected to clinical and orthopedic examination. Animals were confined to stables for at least thirty days, with the same training and feeding routines. Horses with history of lameness, clinical signs of orthopedic, neurological disorders and stereotypic behaviors were excluded from the study. The animals presented a mean age of 3.9 ± 0.8 years, the average approximate height at the withers was 1.6 ± 0.04 m, and the mean approximate body mass was 466.8 ± 39.8 kg. The animals selected for the study are described in Table 1.

**Table 1:** Equine characteristics.

Subject No.	Training regimen	Age (years)	Sex	Approximate body mass (Kg)	Approximate height (m)
1	T1	3	G	410	1.50
2	T1	4	M	425	1.58
3	T1	3	M	430	1.56
4	T1	4	M	470	1.53
5	T1	3	M	445	1.50
6	T1	4	G	440	1.57
7	T1	4	M	400	1.52
8	T1	4	G	520	1.53
9	T1	5	S	495	1.56
10	T1	4	M	455	1.58
11	T2	3	M	430	1.48
12	T2	4	S	460	1.52
13	T2	5	M	485	1.48
14	T2	5	S	500	1.66
15	T2	3	S	490	1.52
16	T2	5	S	500	1.58
17	T2	3	S	450	1.54
18	T2	4	S	460	1.51
19	T2	3	S	545	1.59
20	T2	5	S	525	1.58

Horse description (training regimen [T1= Training on intermittent days; T2= Training on continual days], age and sex [*S*= Stallion; *G*= Gelding; *M*= Mare]) was obtained from each owner and trainer. Approximate body mass and height was acquired using an equine tape.

### Animals housing and management

The animals were housed in individual box stalls measuring 3.4 × 3.4 m, with a double Dutch door and no window. The upper door remained open from 05.00 h at 19:00 h. All horses were fed the similar diets; they were fed in the morning after training at around 09.00 h and in the afternoon at 17.00 h, first with alfalfa hay (approximately 4 kg/ horse/ day), and 1 h later with pelleted concentrate fed (approximately 2.5 kg/ horse/ day) and high-moisture oat groats (approximately 8 kg/ horse/ day), with water *ad libitum*. The amounts of feed and hay were the same in the morning and in the afternoon and the amount of feed and hay offered in the afternoon meal did not allow the horses to have forage or concentrate available for the night time. The animals were fed for at least thirty days with the same routine and no changes were made. The box stalls were bedded with wood shavings, which were cleaned every morning at around 07.00 h and every afternoon at around 15.00 h. After being fed in the afternoon, the horses were kept in the box stalls with the top and bottom doors closed without any human contact until the following morning. The animals were trained on a sand track according to the protocol established by their trainer at around 07.00 h; the training consisted of a walk and trot warm-up followed by a gallop (approximately 500 m walking and trotting; and 1600 m galloping). In the afternoon, the horses were hand-walked for an average duration of 5 min and returned to the stables. All horses were under the care of the same trainer and were under similar training intensity and stall conditions. The environmental conditions during the experiment were: average temperature was 17 °C, the maximum and minimum temperature were respectively 32 °C and 4 °C; the average relative humidity was 81%; the average time of sunrise and sunset was respectively at 07.10 h and at 17.51 h.

### Data collection

The animals were subjected to intermittent (T1) or continual (T2) training. The animals that were submitted to continuous training were exercised in the morning and walked in-hand in the afternoon every day (except on sunday). The animals trained on intermittent days were taken to the sand training track on alternate days and training took place in the morning; in the afternoon, they were walked in-hand. When the horses were not trained, they were taken out of the box stalls for light walks in-hand in the morning and in the afternoon. The time outside the box stalls spent on walking in-hand averaged 5 ± 2 min, whereas the time outside the box stalls spent on training averaged 32 ± 8 min, of which 9 ± 3 min were used for training on the sand track, and the remaining time was to saddling and leading the horses to the track. The night time started at 19.00 h and finished at 05.00 h. In addition to sunrise and sunset, horse management schedules, the time spent in food intake and the arrival of trainers in the stables were taken into account when choosing the night time period. After the animals had already eaten all the food, they were kept in the box stalls with the two double Dutch doors closed and there was no human-animal interaction.

### Accelerometer

A wGT3X-BT® accelerometer version 1.1.0 was used. The data were assessed using ActiLife software. The device weighed 19 gs and measured 4.6 × 3.3 × 1.5 cm. “Cable ties were used to attach the accelerometer to the head collar at the horse's poll” (Morrison et al., 2015).

The accelerometer was set to thirty hertz and programmed to record every movement every five seconds for seventy-two hours. Although the animals remained for seventy-two hours with the monitors recording their physical activity, only the night time without any human-animal interaction and external sounds (workers' conversations, car noises, radio sounds) was analyzed for the study (thirty hours), to minimize the changes in locomotor activity that these interactions could impact. The integrated axes related to longitudinal, lateral, and vertical measurements were assessed, allowing determining the counts per minute (CPM). The following intervals were used for sedentary, light, moderate, and vigorous physical activity levels, respectively: 0–707; 708–1545; 1546–2609; > 2609 CPM, as validated by Morrison et al. (2015).

### Statistical analysis

The variables were analyzed by the SAS Enterprise 9.4 software. The Prism 8 graphpad software was used to create the graphs. Dependent variables included the following: CPM, percentage of time in (sedentary, light, moderate and vigorous) physical activity (%SPA,%LPA,%MPA,%VPA). The independent variables were: training regimen, age and sex. The data were assessed for normality using the PROC UNIVARIATE procedure. All data had normal distribution.

Data were assessed by the PROCGLM procedure (ANOVA), which evaluates unbalanced data, checking interactions between treatments and whether they had a statistical effect on the variables. The interactions analyzed were training regimen, age, and sex by CPM,%SPA,%LPA,%MPA, and%VPA. The means were calculated by Tukey's test using the LSMEANS procedure. The data were expressed as mean and standard deviation. A statistically significant difference was considered when *P* < 0.05, whereas ≤ 0.06 was considered as a tendency.

## Results

Physical activity of intermittently trained (T1) and of continuously trained (T2) horses during the night time is summarized in Table 2. CPM mean showed a higher physical activity (*P* = 0.005) in the intermittent trained animals (516.7 ± 112.3 CPM), in relation with continuously trained animals (346.9 ± 127.2 CPM). The continuously trained animals presented a higher time in sedentary physical activity (85% ± 6.4%), in comparison with intermittent trained (77.4 ± 6.1%; *P* = 0.01). Consequently, intermittently trained horses spent a larger percentage of time in moderate (6.0 ± 2.0%; *P* = 0.01) and vigorous (5.2 ± 1.4%; *P* = 0.002) physical activity, in comparison with continuously trained animals (moderate 3.6 ± 1.8% and vigorous 2.9 ± 1.4% activity). Intermittently trained horses tended to spend a larger percentage of time (11.5 ± 3.0%) in light physical activity, than continuously trained (8.6 ± 3.4%; *P* = 0.06). No effect of age and sex was shown in physical activity during the night time (*P* > 0.05).

**Table 2:** Assessment of night time physical activity (mean ± standard deviation; and 95% confidence intervals [LB = Lower bound; UB = Upper bound]) in horses subjected to training on intermittent days (T1) and continual days (T2).

Variable	T1	LB	UB	T2	LB	UB	P
CPM	516.7 ± 112.3^A^	436.4	597.0	346.9 ± 127.2^B^	255.9	437.9	0.005
% SPA	77.4 ± 6.1^b^	73.0	81.7	85.0 ± 6.4^a^	80.4	89.6	0.01
% LPA	11.5 ± 3.0	9.3	13.6	8.6 ± 3.4	6.1	11.1	0.06
% MPA	6.0 ± 2.0^a^	4.5	7.4	3.6 ± 1.8^b^	2.3	4.9	0.01
% VPA	5.2 ± 1.4^A^	4.2	6.2	2.9 ± 1.4^B^	1.8	3.9	0.002

a, *b* = Values within a row with different superscripts differ significantly at *P* <0.05. A, *B*= Values within a row with different superscripts differ significantly at *P* < 0.01. *P* = significant effect probability. CPM = Counts per minute,%SPA = Percentage of time in sedentary physical activity,%LPA= Percentage of time in light physical activity,%MPA= Percentage of time in moderate physical activity,%VPA = Percentage of time in vigorous physical activity.

## Discussion

The author´s hypothesis that intermittently trained animals had a higher level of night time physical activity in comparison to continual training was supported. Continuously trained animals spent a longer time in sedentary physical activity in the night time; therefore, they spent more time at rest. Intermittently trained animals spent most of the time in light, moderate, and vigorous physical activity when compared to the other group; this does not mean that these animals trotted or cantered [CPM cutoff points for moderate and vigorous physical activity established by Morrison et al. (2015)] inside the box stalls during the night, but it does indicate that these animals were more restless during the night time.

Increased physical activity has been associated with confinement (Giannetto et al., 2016; Piccione et al., 2008). Stabled horses that were exercised changed their nocturnal behavior, in comparison with non-exercised ones (Krzak, Gonyou & Lawrence, 1991), as was observed in the present study. Therefore, the exercise probably has several effects on the horses, physiologically as well as psychologically, carrying to a higher degree of physical weariness and contributing to horse balance (Redbo, Redbo-Torstensson, Ödberg, Hedendahl & Holm, 1998). Probably higher energy expenditure during training may have resulted in a better night's rest. In human athletes, self-reported sleepiness at bedtime is greater on nights of high-intensity training (Robey et al., 2013). These findings are also in line with those found by Houpt et al. (2001), who pointed out that horses that did not train every day had a compensatory increase in locomotion when allowed to roam free, indicating a response to exercise deprivation. Therefore, the training regimen is a factor that influences the nocturnal behavior of athlete horses, and it is an aspect that ethologically should not be underestimated.

Nocturnal locomotion has been evaluated commonly in elite human athletes using accelerometers, being a good tool to qualify night time sleep and rest (Leeder, Glaister, Pizzofero, Dawson & Pedlar, 2012; Mah, Mah, Kezirian & Dement, 2011; Robey et al., 2013; Sargent, Lastella, Halson & Roach, 2014). Leeder et al. (2012) used accelerometers to determine the influence of gender in the sleep quality of human olympic athletes and found that women have a better sleep quality. Other studies showed that males are more active during the whole day than females (Belcher et al., 2010; Troiano et al., 2008). Sex impact was not observed in locomotion during the night time in racehorses in this study as it was in human athletes, although it is important to emphasize that nocturnal locomotion and the sleep patterns of horses and humans are different. In addition, it was showed in humans an age-related decline in physical activity, particularly from childhood through adolescence (Belcher et al., 2010; Troiano et al., 2008) however, at least at the age of 3 to 5 years in horses, no differences were observed between ages. A limitation of the present study was had a small group of horses to make inferences on sex an age. In addition, age and sex were not evenly distributed between groups, because the main objective of the study was to evaluate the effect of training regimen; and age and sex were considered only.

The head was chosen for attachment of the accelerometer as owners acceptance is high at this site, the monitor has optimal sensitivity and specificity, besides is less prone to fall off during vigorous physical activity (Morrison et al., 2015). There were no monitors-related problems during the study.

## Conclusion

This study demonstrated that horses on continual training have lower physical activity in the night time than those that train on intermittent days. Also, we report an area for future study with use of accelerometers as a tool to monitor physical activity during the night.

## Ethical considerations

The study protocol was previously analyzed and approved by the Animal Ethics Committee of Universidade Federal do Rio Grande do Sul (process no. 33,440), in accordance with the National Council for the Control of Animal Experimentation.

## Autorship statement

A. S. Rumpel, M. M. Alievi and J. O. Jardim Filho developed the idea for the article. A. S. Rumpel performed data collection and processing. Data analysis by A. S. Rumpel and C. A. Camacho-Rozo. Data interpretation by A. S. Rumpel, M. M. Alievi, L. A. H. Schuster, A. V. Silva and M. P. Ferreira. All authors contributed to critical revision, written and approved the final manuscript.

## Ethical statement

The study protocol was previously analyzed and approved by the Animal Ethics Committee of Universidade Federal do Rio Grande do Sul (process no. 33,440), in accordance with the Council for International Organization of Medical Sciences. The animals were included in the study after the owners read and signed a free informed consent form. No changes were made to the training and feeding routine of all animals.

## Declaration of Competing Interest

None of the authors of this paper have a financial or personal relationship with other people or organizations that could inappropriately influence or bias the content of the paper. It is to specifically state that “no competing interests are at stake and is no conflict of interest” with other people or organizations that could inappropriately influence or bias the content of the paper. This research did not receive any specific grant from funding agencies in the public, commercial, or not-for-profit sectors.
